# Fatigue Status in Relation to Lifestyle in Healthy Japanese Adolescents

**DOI:** 10.1155/2010/520320

**Published:** 2010-09-19

**Authors:** Ning Zou, Masaru Kubota, Eriko Kuruma, Chiaki Kojima, Ayako Nagai

**Affiliations:** Department of Human Life and Environment, Nara Women's University, Kitauoya-nishimachi, Nara 630-8506, Japan

## Abstract

In order to investigate the prevalence of physical, mental, and chronic fatigue syndrome-(CFS-) related fatigue and its relation to lifestyle, 1,225 adolescents (591 males, 634 females) aged 11 to 16 years were asked to complete a self-reported questionnaire on fatigue status and lifestyle in the past one month. There was no gender difference in physical and mental fatigue scores, but CFS-related scores were significantly higher in females than in males. These scores were found to increase with the increase of age. After adjusting for age and gender, multiple regression analysis showed that physical and mental fatigue scores were associated with sleeping hours, extracurricular sports activity, food balance, the frequencies of snacks between regular meals, intake of sugar-sweetened beverages, and visits to the nurse's room. This paper is the first large cross-sectional study on fatigue in healthy adolescents in Japan, albeit there were numerous such studies in Western countries.

## 1. Introduction

Easy fatigability is a problem commonly seen in modern society [1]. Among fatigue-related disorders, chronic fatigue syndrome (CFS), which is defined by intractable physical and mental fatigue lasting more than 6 months with a number of accompanying symptoms, has been well investigated [2, 3]. In adults, several surveys have indicated the prevalence of CFS in the community as 0.007–2.54% [1, 4–6]. Generally, women tend to have a higher prevalence rate than men [1, 4–6]. On the other hand, the prevalence of CFS in adolescents is roughly estimated to be 0.07–0.5% [7–9]. CFS has also been reported in children aged 11 years and younger [10].

 There are several studies on fatigue in populations of healthy adolescents and children in Western countries. These studies have demonstrated a wide variety of prevalence depending upon the study population and definition of fatigue [8, 9, 11, 12]. For example, the incidence of chronic fatigue in a general population of 842 British adolescents was 0.4%–1.1%. Chronic fatigue in this paper was defined as severe fatigue that was not helped by rest and lasted at least 6 months [8]. In a population-based study of 8,586 US adolescents, 138 (1.6%) had fatigue for ≥one month, and 107 (1.2%) had chronic fatigue for ≥6 months [11]. Here, fatigue was judged by information from the telephone interview with a parent or guardian. A recent Dutch study indicated that 6.5% of boys and 20.5% of girls had severe fatigue, whose checklist individual strength (CIS) scores were above the cutoff point during the last 2 weeks [12]. Notably, chronic fatigue is known to be associated with symptoms of depression or anxiety and with future occurrence of chronic fatigue syndrome in these reports [1, 12, 13]. Reports about the actual conditions of fatigue in healthy Asian adolescents are quite limited. As the only report in Asian countries, to our knowledge, Okamoto et al. investigated the characteristics of fatigue in association with lifestyle in 247 healthy Japanese adolescents [14]. Although the fatigue scores were not evaluated, they found high frequencies of complaints of “drowsiness and dullness” and “difficulty in concentration”. However, their study included only a limited number of subjects, and it was carried out more than ten years ago. Therefore, we tried to investigate the present fatigue status in healthy Japanese adolescents, paying special attention to their lifestyle. 

## 2. Materials and Methods

### 2.1. Subjects

This study was carried out in Nara city in the spring of 2008 with 1,225 participants. Nara is an urban city in Japan with a population of approximately 370,000. Among them, the approximate population of children and adolescents aged between 3 and 17 years was 45,000. The mean annual income of each household was almost similar to the average of Japan (2006, census). The participants included 421 students in the 6th grade of elementary school (11 years), 226 in the 1st grade (12 years) and 243 in the 3rd grade (14 years) of junior high school, and 335 in the 2nd grade of senior high school (16 years). There were 591 males and 634 females. At school, they were asked to complete a questionnaire about their fatigue status and lifestyles on average over the past one month. We obtained consent from all students beforehand for using their data in our study. In addition, their parents were informed of this survey beforehand. This project was approved by the ethical and epidemiological committee at Nara Women's University.

### 2.2. Questionnaire

We devised our own questionnaire as shown in the appendix. There were 4 questions each on physical and mental fatigue. Each question was scored from 0 to 3: 0, not at all; 1, a few times per month; 2, a few times per week; 3, almost every day. The scores given by the participant were then summed to calculate physical and mental fatigue scores separately (range: 0–12). Subjects were also asked about seven CFS-related elements based on the diagnostic criteria of the disease [3], hereafter defined as the CFS-related score. The CFS-related score was determined after scoring these elements and summing them up (range: 0–21). Questions about lifestyle were as follows: (1) sleep: daily sleep hours (<7 hours; score 0, or ≥7 hours; score 1), (2) sports: extracurricular sports activity more than one hour/day (every day; score 1, few times a week; score 2, once a week; score 3, and rarely; score 4), (3) food: snacks between regular meals (every day; score 1, few times a week; score 2, once a week; score 3, and rarely; score 4), and intake of sugar-sweetened beverages (every day; score 1, sometimes; score 2, and rarely; score 3), and food intake of (a) milk products, (b) brightly colored vegetables, (c) eggs, (d) fish or meat, and (e) beans or legumes (every day; score 3, few times a week; score 2, and rarely; score 1). Scores of each element were summed to calculate the “food balance score” (range: 5–15). Whether the students visited the nurse's room at school because of fatigue or tiredness was also asked. The nurse's room is the room where a nurse or a teacher in charge of health problems at school is stationed. Body mass index (BMI) was calculated from the height and weight measured at a regular school checkup.

### 2.3. Evaluation of the Validity and the Reliability of the Questionnaire

Before starting the survey, we have checked the validity and the reliability using 100 female students aged 18–20 years at Nara Women's University. They were asked to answer anonymously our questionnaire on their fatigue and Chalder's scale simultaneously. The items of the original Chalder's scale were translated into Japanese by the chief investigator, M. Kubota. 

### 2.4. Statistics

Differences in mean physical and mental fatigue, and CFS-related scores according to gender and age were examined by Student's *t*-test and one-way analysis of variance (ANOVA), respectively. The association of these scores with various lifestyle elements and BMI was evaluated by multiple regression analysis. The total population was divided into two groups by the total fatigue score (physical plus mental fatigue score), and association with demographic factors, lifestyles, visit to the nurse's room or BMI was estimated by logistic regression analysis. A statistical analysis was made by “StatMate IV” (ATMS, Tokyo, Japan). *P*-values less than .05 were considered significant.

## 3. Results

### 3.1. Reliability and Validity of Our Questionnaire

Since we have devised our own questionnaire, we first evaluated the reliability and the validity of the questionnaire in female students at our university. Cronbach's *α*s for physical, mental, and total fatigue scores were 0.76, 0.72, and 0.83, respectively. These values (i.e., > 0.7) are considered to be good for the internal consistency and to satisfy the reliability. Next, we evaluated the reliability of our questionnaire in comparison with the same subscales in the Chalder's scale, which was already validated. Correlation coefficients were 0.76 for physical fatigue scores, 0.64 for mental fatigue scores, and 0.78 for physical + mental fatigue scores. This fact supports the construct validity of our questionnaire.

### 3.2. Physical Fatigue, Mental Fatigue, and CFS-Related Scores in Relation to Age and Gender

Subject numbers according to age and gender are shown in Table 1. Table 1 also indicates the mean and 95% confidence interval (CI) of physical fatigue, mental fatigue, and CFS-related scores for different ages and gender. Significant increases of these scores with age were found in the male, female, and total populations (*P* < .01). There was no gender difference in physical and mental fatigue scores, whereas CFS-related scores were significantly higher in females except for 12-year-olds. In the present participants, Cronbach's *α*s for physical, mental, and total fatigue scores were 0.76, 0.74, and 0.85, respectively. This fact also showed the good internal consistency.

### 3.3. Association between Physical Fatigue, Mental Fatigue, CFS-Related Scores and Various Lifestyles or BMI

By multiple regression analysis after adjusting for age and gender, these scores were found to be associated with all lifestyles except for snacks between meals in CFS-related scores (Table 2). Shorter duration of sleep, less extracurricular sports activity, lower food balance score (i.e., less balanced diet), and higher frequency of snacks or sugar-sweetened beverages were predictors of increased scores. In contrast, BMI did not show any significant association in each score.

### 3.4. Association of Physical plus Mental Fatigue Scores with Demographic Factors, Lifestyles, or BMI

In order to evaluate the total quality of life, we have summed up the physical and mental fatigue scores as the total fatigue score. Then, the total population was divided into two groups according to the total fatigue score, that is, low fatigue (scores 0–12) and high fatigue (scores 13–24). Logistic analysis was carried out to determine associations of either group with demographic factors, lifestyle, visits to the nurse's room or BMI. As shown in Table 3, age (*P* < .001), sleep duration (*P* < .001), food balance score (*P* < .001), frequencies of snacks and sugar-sweetened beverages (*P* < .05), and visits to the nurse's room (*P* < .001) revealed significant association with the total fatigue score.

## 4. Discussion

Fatigue in adolescents has been an important issue of research in recent decades. There are objective markers newly invented for evaluation of fatigue [15]. Although a self-reported questionnaire has the weakness of not being objective, it is still thought to be the most reliable and acceptable method of collecting activity data from a large population of adolescents [16]. Among various self-reported questionnaires [17–19], the CIS score, which was originally developed for adults, has been most widely used [18]. The CIS consists of 20 questions with 4 subclasses, that is, fatigue severity, concentration, reduced motivation, and physical activity. The CIS asks the fatigue status over the preceding two weeks. The validity of CIS scores has been established [3], and the use of CIS scores makes it possible to make comparisons among different data. Another popular questionnaire, Chalder's scale, consists of 14 elements and separates physical and mental symptoms [17]. Chalder's scale is simple, and thereby is useful in large community samples. 

 In the present study, we devised our own questionnaire, since the nuance of some questions in the previously validated questionnaire such as “Do you feel weak?” is difficult to be translated into Japanese accurately. In addition, those questionnaires have their own disadvantages. Since the CIS questionnaire consists of seven scales for each question, it is rather difficult for children to find an appropriate answer [12]. Inclusion of several symptoms closely related to CFS is the disadvantage of Chalder's scale. In our questionnaire, most elements appearing in the physical and mental fatigue questions except for those in mental fatigue, 3 and 4, were drawn from the CIS with modification [18]. As for CFS-related symptoms, we drew 6 elements from the report of the international CFS study group [3]. Although exact comparison with previous studies is thus difficult, our study presents several issues. First, there was no gender difference in either physical or mental fatigue showing a contrast with previous studies demonstrating female dominance [8, 9]. The reason for this disparity is uncertain at present, but one possible explanation is that Japan is a male-dominated society, and that boys are obliged to study hard at school from early childhood. On the other hand, CFS-related scores were significantly higher in females. Notably, the order of the prevalence among 6 CFS-related symptoms was almost identical to that in the previous study (data not shown) [13]. Second, we did not investigate the rate of school absence, but self-reported visits to the nurse's room at school because of fatigue or tiredness increased with an increase of fatigue severity. This fact should be further investigated, since severe or chronic fatigue is thought to disrupt daily life, for example resulting in school absence [20]. Finally, various lifestyle factors were found to be closely associated with fatigue. Among them, the association of unhealthy dietary habits with fatigue is consistent with previous studies [14]. However, the role of sleep duration, sports activity or degree of obesity differs from study to study, presumably due to differences in study design [9, 12, 14].

 There are a number of limitations in the present study. First, as mentioned above, we developed and used our own questionnaire. This makes it difficult to compare our results with those of previous studies. Second, this is a cross-sectional study done in a single period of spring, 2008. Since the degree of fatigue in an individual may change among different seasons of the year, a longitudinal study for at least one year is warranted [9]. Finally, we did not ask anything about personal medical histories from the viewpoint of protecting personal information. In fact, it is very difficult to ask the question such as “Do you have any chronic disease”, even if it is anonymous. Therefore, there is a possibility that some students, especially those with severe fatigue, may have underlying disorders. 

 In conclusion, we carried out the large cohort study on the present fatigue status in healthy Japanese adolescents. The finding that there was not any significant gender difference in both physical and mental fatigue scores should be paid attention considering the female dominance in previous reports from Western countries. This might be partly explained by the inherent cultural background in Asian countries. However, generalization of our data directly to other Asian counties should be cautious. In spite of the limitations, we still believe that our findings will be useful for understanding the similarities and differences of fatigue among healthy adolescents in different developed countries. A further followup study to investigate whether chronic or severe fatigue in adolescents is connected with CFS or CFS-like illness in later life is necessary.

## Figures and Tables

**Table 1:** Subject number, physical fatigue score, mental fatigue score, and CFS-related score.

		Subject number	Physical fatigue score	Mental fatigue score	CFS-related score
11 yr	Total	421	5.0 [4.7–5.2]^†^	3.5 [3.2–3.8]	5.2 [4.8–5.5]
	Male	204	4.9 [4.5–5.3]	3.3 [2.9–3.8]	4.9 [4.4–5.3]*
	Female	217	5.0 [4.6–5.4]	3.6 [3.2–4.0]	5.5 [4.9–5.9]

12 yr	Total	226	5.5 [5.1–5.8]	4.0 [3.6–4.4]	5.0 [4.6–5.5]
	Male	109	5.7 [5.1–6.2]	4.0 [3.5–4.6]	5.1 [4.4–5.7]
	Female	117	5.3 [4.8–5.8]	3.9 [3.4–4.4]	5.0 [4.5–5.6]

14 yr	Total	243	6.5 [6.2–6.9]	6.0 [5.6–6.4]	6.9 [6.5–7.4]
	Male	118	6.3 [5.8–6.7]	5.8 [5.2–6.3]	6.1 [5.4–6.7]**
	Female	125	6.8 [6.3–7.3]	6.2 [5.7–6.8]	7.8 [7.2–8.4]

16 yr	Total	335	6.6 [6.3–6.9]	6.0 [5.7–6.3]	6.5 [6.1–6.8]
	Male	160	6.5 [6.1–7.0]	5.7 [5.2–6.3]	6.0 [5.6–6.5]**
	Female	175	6.7 [6.3–7.0]	6.3 [5.9–6.7]	6.8 [6.4–7.3]

All	Total	1225	5.8 [5.6–6.0]	4.8 [4.6–4.9]	5.8 [5.6–6.0]
	Male	591	5.8 [5.5–6.0]	4.6 [4.3–4.9]	5.4 [5.2–5.7]**
	Female	634	5.9 [5.6–6.1]	4.9 [4.7–5.2]	6.2 [5.9–6.5]

^†^Numbers in parentheses show 95% CI.

Differences between male and female subjects at each age are: *P < .05, **P < .01, no marks; not significant

**Table 2:** Multiple regression analysis between physical fatigue, mental fatigue, CFS-related scores, and lifestyles or BMI after adjusting for age and gender.

	Physical fatigue score		Mental fatigue score		CFS-related score	
	*β* coefficient	95% CI	*β* coefficient	95% CI	*β* coefficient	95% CI
Lifestyles^†^						
Sleeping Hours	−1.04	−1.39 ~ −0.69**	−1.39	−1.76 ~ −1.03**	−1.58	−1.98 ~ −1.12**
Extracurricular sports activity	0.25	0.12 ~ 0.39**	0.17	0.03 ~ 0.32*	0.19	0.04 ~ 0.35*
Food balance score	−0.24	−0.32 ~ −0.15**	−0.29	−0.39 ~ −0.21**	−0.21	−0.31 ~ −0.11**
Snacks between regular meals	−0.17	−0.34 ~ −0.01*	−0.22	−0.39 ~ −0.05*	−0.18	−0.37 ~ 0.02
Sugar-sweetened beverage	−0.45	−0.70 ~ −0.19**	−0.44	−0.71 ~ −0.17**	−0.88	−1.36 ~ −0.41**
BMI	0.06	−0.03 ~ 0.15	0.07	−0.02 ~ 0.16	0.07	−0.04 ~ 0.17

*P < .05,**P < .001

^†^For the analysis, scores were given for each element. The details are shown in “Materials" section.

**Table 3:** Multiple logistic regression analysis between total scores and demographic or lifestyle.

Total scores^†^	0–12	13–24	*β* coefficient	Odds ratio [95% CI]	*P*-value
Total number	786	439			
Gender			0.151	1.16 [0.89–1.52]	.27
Male	392 (49.9) ^‡^	199 (45.3)			
Female	394 (50.1)	240 (54.7)			
Age			0.191	1.21 [1.13–1.29]	<.001
11 yr	326 (41.5)	95 (21.6)			
12 yr	165 (21.0)	61 (13.9)			
14 yr	127 (16.1)	116 (26.4)			
16 yr	168 (21.4)	167 (38.1)			
Sleep duration			−0.799	0.45 [0.34 − 0.59]	<.001
<7 hrs	296 (37.7)	290 (66.1)			
≥7 hrs	490 (62.3)	149 (33.9)			
Extracurricular sports activity			0.089	1.09 [0.98–1.21]	.087
Every day	383 (48.7)	179 (40.8)			
Few times/week	128 (16.3)	60 (13.7)			
Once/week	103 (13.1)	50 (11.3)			
Rarely	172 (21.9)	150 (34.2)			
Food balance score	12.6 [12.4–12.7] ^ §^	12.1 [11.8–12.2]	−0.152	0.86 [0.80–0.92]	<.001
Snacks between regular meals			−0.137	0.87 [0.77–0.98]	<.05
Every day	59 (7.5)	52 (11.9)			
Few times/week	85 (10.8)	54 (12.3)			
Once/week	133 (16.9)	85 (19.4)			
Rarely	509 (64.8)	248 (56.4)			
Sugar-sweetened beverage			−0.223	0.79 [0.66–0.97]	<.05
Every day	79 (10.1)	75 (17.1)			
Sometimes	404 (51.4)	218 (49.7)			
Rarely	303 (38.5)	146 (33.2)			
Visits to the nurse's room			0.767	2.15 [1.51–3.07]	<.001
No	701 (89.2)	348 (79.3)			
Yes	85 (10.8)	91 (20.7)			
BMI	18.6 [18.3–18.9] ^ §^	19.4 [18.8–19.8] ^ §^	0.018	1.02 [0.95–1.09]	.62

^†^total scores = physical fatigue scores + mental fatigue scores

^‡^ Numbers in parentheses indicate the percentages

^§^ Mean and 95% CI are shown.

## Appendix

Please score elements from 0 to 3 in boxes as the average for the past one month. (0: not at all, 1: a few times per month, 2: a few times per week, and 3: almost every day).

- * Physical Fatigue-Related Elements *
  1. ( ) How often do you feel tired?
  2. ( ) How often do you feel to take more rest?
  3. ( ) How often do you feel forceless?
  4. ( ) How often do you feel that you are in a physically bad condition?
- *Mental Fatigue-Related Elements *
  1. ( ) How often do you feel you are making careless mistakes?
  2. ( ) How often do you feel a lack energy to do something?
  3. ( ) How often do you feel depressed?
  4. ( ) How often do you feel irritated?
- *CFS-Related Elements *
  1. ( ) How often do you experience a feverish feeling?
  2. ( ) How often do you experience unrefreshing sleep?
  3. ( ) How often do you experience headaches?
  4. ( ) How often do you experience muscle pain (not right after sports)?
  5. ( ) How often do you experience joint pain (not right after sports)?
  6. ( ) How often do you experience sore throat?
  7. ( ) How often do you experience difficulty of concentration?
